# Genome-Wide Characterization of Extrachromosomal Circular DNA in the Midgut of BmCPV-Infected Silkworms and Its Potential Role in Antiviral Responses

**DOI:** 10.3390/ijms26020818

**Published:** 2025-01-19

**Authors:** Xinyu Tong, Chao Lei, Yilin Liu, Mei Yin, Huan Peng, Qunnan Qiu, Yongjie Feng, Xiaolong Hu, Chengliang Gong, Min Zhu

**Affiliations:** School of Life Sciences, Soochow University, Suzhou 215123, China

**Keywords:** eccDNAs, BmCPV, mRNA, miRNA, circRNA

## Abstract

Extrachromosomal circular DNAs (eccDNAs) has been found to be widespread and functional in various organisms. However, comparative analyses of pre- and post-infection of virus are rarely known. Herein, we investigated the changes in expression patterns of eccDNA following infection with *Bombyx mori* cytoplasmic polyhedrosis virus (BmCPV) and explore the role of eccDNA in viral infection. Circle-seq was used to analyze eccDNAs in the midgut of BmCPV-infected and BmCPV-uninfected silkworms. A total of 5508 eccDNAs were identified, with sizes varying from 72 bp to 17 kb. Most of eccDNAs are between 100 to 1000 bp in size. EccDNA abundance in BmCPV-infected silkworms was significantly higher than in BmCPV-uninfected silkworms. GO and KEGG analysis of genes carried by eccDNAs reveals that most are involved in microtubule motor activity, phosphatidic acid binding, cAMP signaling pathway, and pancreatic secretion signaling pathways. Several eccDNAs contain sequences of the transcription factor SOX6, sem-2, sp8b, or Foxa2. Association analysis of eccDNA-mRNA/miRNA/circRNA revealed that some highly expressed genes are transcribed from relevant sequences of eccDNA and the transcription of protein coding genes influenced the frequency of eccDNA. BmCPV infection resulted in changes in the expression levels of six miRNAs, but no known miRNAs with altered expression levels due to changes in eccDNA abundance were identified. Moreover, it was found that 1287 and 924 sequences representing back-spliced junctions of circRNAs were shared by the junctions of eccDNAs in the BmCPV-infected and uninfected silkworms, respectively, and some eccDNAs loci were shared by circRNAs on Chromosomes 2, 7, 11, 14, and 24, suggesting some eccDNAs may exert its function by being transcribed into circRNAs. These findings suggest that BmCPV infection alter the expression pattern of eccDNAs, leading to changes in RNA transcription levels, which may play roles in regulating BmCPV replication. In the future, further experiments are needed to verify the association between eccDNA-mRNA/miRNA/circRNA and its function in BmCPV infection.

## 1. Introduction

Circular DNA occurs commonly in nature, particularly in bacteria and yeast, where it often occurs within bacterial plasmids or mitochondrial DNA. Extrachromosomal circular DNA (eccDNA), closed circular DNA that originates from chromosomes but exists independently of chromosomal DNA, can be either single- or double-stranded. Although eccDNA was observed using electron microscopy as early as 1965 [1], it was thought to represent garbage fragments of DNA outside of chromosomes and attracted little research attention. It is now known that eccDNA occurs widely within cells, and that various eukaryotic chromosomes such as yeast [2], nematodes [3], *Drosophila* [4], pigeons [5], *Oxytricha* [6], humans [7,8,9], and plants [10,11,12] can form it. EccDNA can increase oncogene copy number by carrying them [13], promote their amplification through autonomous replication [14], and enhance their plasticity and instability [15,16]. The latest research indicates that eccDNA can function as a mobile enhancer to modulate gene expression [17]. Additionally, it has been discovered that eccDNA possesses autonomous transcriptional activity, regulating gene expression by producing RNA [18]. Despite this, few studies have examined changes in organisms before and after viral infection. Moreover, the function of host eccDNA in the context of viral infection remains unclear.

*Bombyx mori* cytoplasmic polyhedrosis virus (BmCPV) belongs to the Reoviridae family and is a representative species of cypovirus. The polyhedra are digested by alkaline digestive juices in the lumen of silkworm’s midgut, releasing virus particles that infect midgut cells, resulting in onset of cytoplasmic polyhedrosis and adversely affecting sericulture [19]. While historically the way in which viruses and hosts interact has focused on studying the regulation of gene expression, the function of molecules such as coding proteins of *B. mori* [20], miRNAs [21,22], lncRNAs [23], circRNAs [24,25], and others in the interaction between BmCPV and *B. mori* have been recently investigated. However, there have been no studies on how BmCPV infection affects eccDNA.

In this study, we analyzed the characteristics of eccDNAs in the midgut of silkworms infected with BmCPV and those uninfected utilizing the Circle-seq. The analysis revealed a total of 5508 eccDNAs, varying in size from 72 bp to 17 kb, the majority falling between 100 to 1000 bp. Motif analysis revealed that repeat nucleotide patterns are located at the 5′ and 3′ ends of the eccDNA breakpoints. These motifs may contribute to several possible models regarding the generation of eccDNA. The analysis of genes carried by eccDNAs through GO and KEGG revealed that most of these eccDNAs participate in microtubule motor activity, phosphatidic acid binding, cAMP signaling pathway, pancreatic secretion signaling pathways, which may play a regulatory role on BmCPV infection through these pathways. We found that many eccDNAs contain sequences of transcription factor-related genes, indicating that these eccDNAs may have the capacity to transcribe both full-length and truncated genes and cooperate to regulate BmCPV infection. Furthermore, BmCPV infection increased the abundance and quantity of eccDNA in the midgut, some highly expressed genes were found to be transcribed from relevant sequences of eccDNA, but no known miRNAs with altered expression levels due to changes in eccDNA abundance were identified. Moreover, 1287 and 924 sequences representing back-spliced junctions of circRNAs were shared by the junctions of eccDNAs in the BmCPV-infected and uninfected silkworms, respectively, some eccDNAs loci were shared by circRNAs on Chromosomes 2, 7, 11, 14, and 24, suggesting some eccDNAs may exert its function by being transcribed into circRNAs. These findings suggested that BmCPV infection alter the expression pattern of eccDNAs in the silkworm midgut, leading to changes in RNA transcription levels, which may play roles in regulating BmCPV replication.

## 2. Results

### 2.1. Properties of eccDNA from Silkworm Midguts

**Figure 1:**
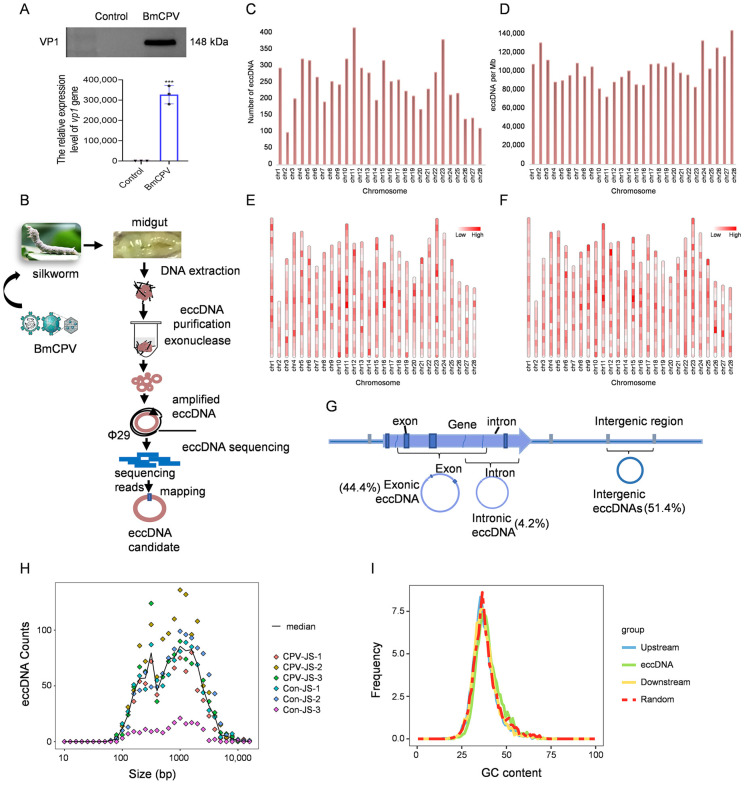
Characteristics of eccDNAs in midguts of *B. mori*. (**A**) Validation of BmCPV-infected silkworms. (0.0001< *** *p* < 0.001, n = 3). (**B**) A schematic overview of the Circle-Seq approach applied for genome-wide profiling of eccDNA from the silkworm midgut. (**C**) The quantity of eccDNAs across various chromosomes. (**D**) The number of formed eccDNAs per Mb on each chromosome. (**E**,**F**) Heatmap of the distribution of eccDNAs in silkworm midguts on the chromosome in BmCPV-uninfected (**E**) and BmCPV-infected silkworms (**F**). (**G**) The distribution of eccDNA among different genomic elements. (**H**) Distribution of sizes for eccDNA in BmCPV-infected and uninfected silkworm midguts. (**I**) Comparison of GC content in the eccDNA locus and its adjacent upstream and downstream regions to the genomic average.

To determine whether BmCPV infected the silkworm, the expression level of VP1 after virus infection was measured by Western blot and qPCR. The results demonstrated that VP1 expression level increased significantly after virus infection, indicating that the silkworms were infected with BmCPV (Figure 1A). Circle-seq was used to investigate the eccDNAs in the silkworm midgut (Figure 1B). The numbers of clean reads for BmCPV-infected (CPV-JS) and BmCPV-uninfected (Con-JS) silkworm midguts were 221,622,188, 196,398,878, 153,274,728, 148,146,262, 146,211,074, and 137,778,670, respectively (Table 1). Following the filtering of low-quality data with Circle-Map, we identified 5508 eccDNAs in total (Circle score > 0). The mapping of eccDNAs in the genome was conducted based on their originating chromosomes. An analysis of the number of eccDNAs formed on each chromosome revealed that chromosome 11 forms 336 eccDNAs, with the highest number, while chromosome 2 forms 79 eccDNAs, with the lowest number (Figure 1C). Additionally, chromosome 28 produced the highest number of eccDNAs per MB at 120,933, whereas chromosome 11 produced the lowest, with 61,266 eccDNAs per MB (Figure 1D). These eccDNAs were detectable on all chromosomes, but the hotspots that generate eccDNA vary across each chromosome. (Figure 1E,F). Based on their localization, eccDNAs were classified as either intergenic (51.4%) or genic (48.6%), the latter mostly derived from exons (Figure 1G). The size range of these eccDNAs was from 72 bp to 17 kb, although most were between 100 and 1000 bp (Figure 1H). There was similar enrichment of GC contents in eccDNA sequences of silkworm midgut compared with other genomic regions (Figure 1I). This is inconsistent with previous reports that high GC content is a typical characteristic of eccDNAs [26].

### 2.2. Verification of Silkworm eccDNAs

**Figure 2:**
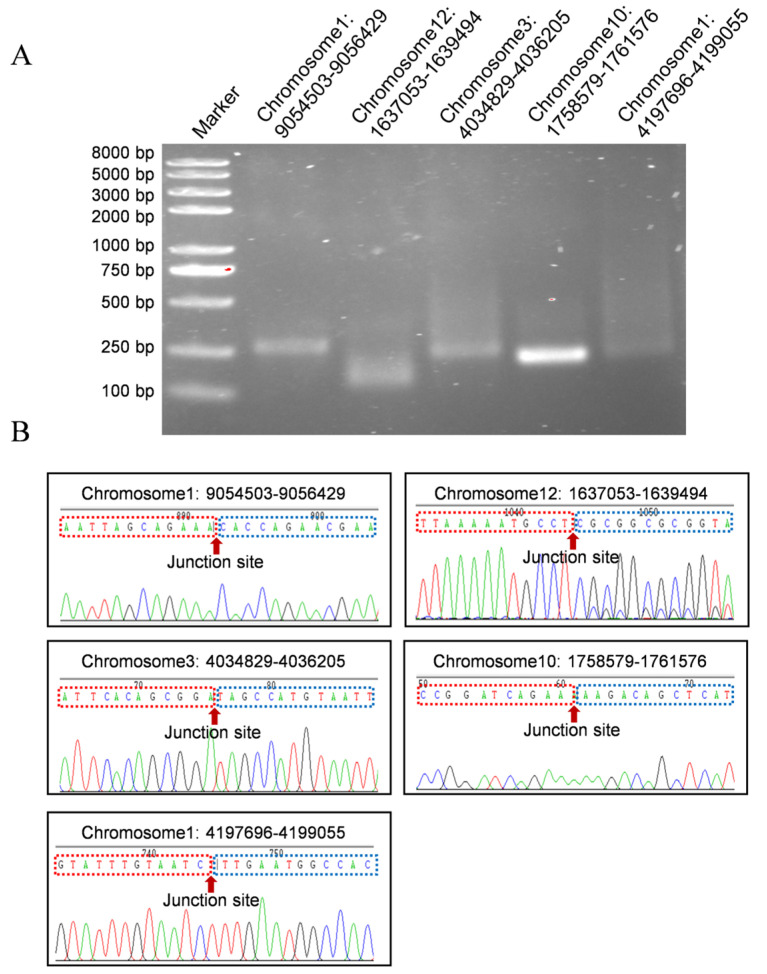
Validation of eccDNAs. (**A**) For each predicted eccDNA, divergent PCR primers were designed to specifically amplify the circular molecules and their junction sites. Five eccDNAs were chosen for additional validation (including Chromosome 1: 9054503–9056429, Chromosome 12: 1637053–1639494, Chromosome 3: 4034829-4036205, Chromosome 10: 1758579–1761576 and Chromosome 1: 4197696–4199055). (**B**) PCR products validation by Sanger sequencing. Arrow indicated junction site. The red arrow indicates the junction site of eccDNAs.

Five eccDNAs were randomly selected for validation using divergent primers. Products of the expected size were successfully amplified which were confirmed by Sanger sequencing (Figure 2). The selected eccDNAs were found to be covalently closed, which aligns with the Circle-seq results.

### 2.3. The Motifs Flanking the Breaking Points of eccDNA Are Conserved

**Figure 3:**
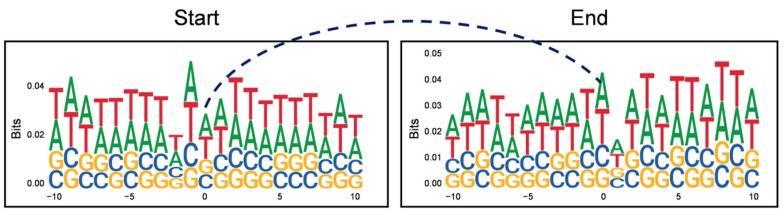
Motif analysis of the sequences flanking the breaking points of eccDNAs. The picture on the left represents the motif of the 10 bp sequences located upstream and downstream of the 5′ breaking points of eccDNA. The picture on the right represents the motif of the 10 bp sequences located upstream and downstream of the 3′ breaking points of eccDNA.

To understand the generation of eccDNA, we examined the nucleotide composition of 10 bp on either side of the 5′ and 3′ breaking points of each eccDNA. It is noteworthy that repeat nucleotide patterns rich in AT were detected in the sequences adjacent to both the 5′ and 3′ breaking points (Figure 3).

### 2.4. Profile of Differential Expression of eccDNAs in the Midguts of Silkworms

**Figure 4:**
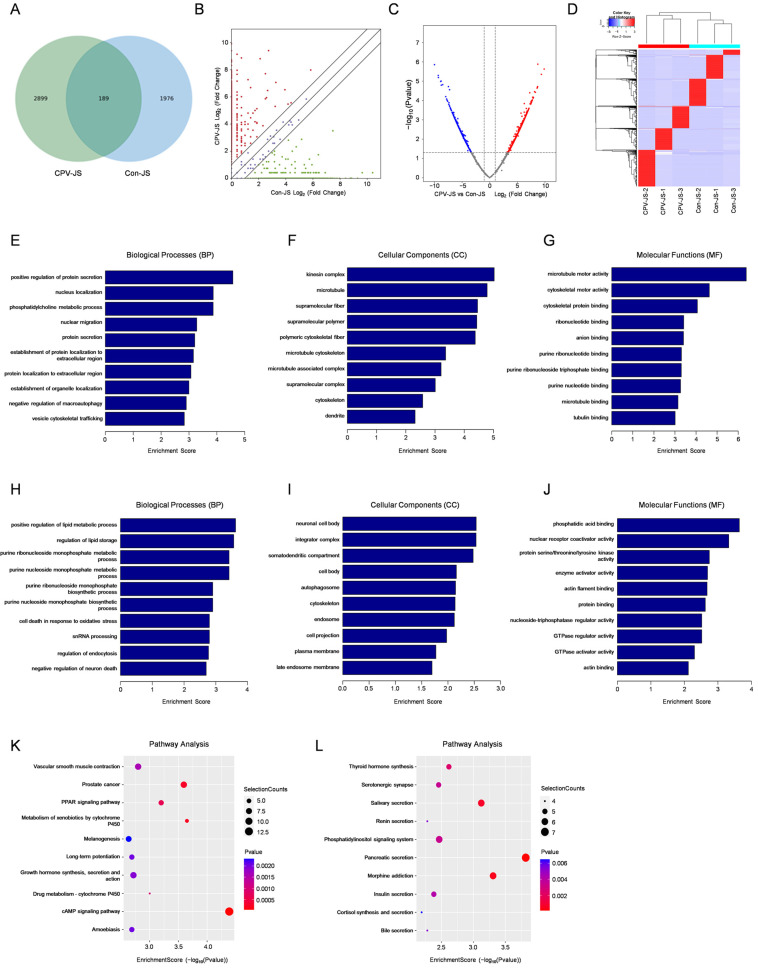
Expression patterns of ecccDNA in BmCPV-infected and uninfected midgut and functional annotation of differentially expressed eccDNAs. (**A**) The quantity of eccDNA co-expressed in BmCPV-infected and uninfected silkworm midguts. CPV-JS represents BmCPV-infected group. Con-JS represents BmCPV-uninfected group. (**B**) The scatter plot of differentially expressed eccDNAs. (**C**) The volcano plots of differentially expressed eccDNAs. (**D**) Heat map and hierarchical clustering of eccDNAs. (**E**–**J**) The biological processes, cellular components, and molecular functions are associated with the up-regulated (**E**–**G**) and down-regulated (**H**–**J**) eccDNAs. (**K**,**L**) Analysis of KEGG pathways related to the up-regulated (**K**) and down-regulated (**L**) eccDNAs.

Features of the distribution of eccDNAs varied between BmCPV-infected and BmCPV-uninfected midgut samples. According to the Venn diagram, of the 5064 eccDNAs, 2899 were detected solely in BmCPV-infected samples, 1976 were found only in BmCPV-uninfected samples, and 189 eccDNAs were present in both groups (Figure 4A). Consequently, the level of eccDNA expression in BmCPV-infected and BmCPV-uninfected midguts were compared. Based on the screening criteria, 2059 eccDNAs was defined as differentially expressed eccDNAs, including 1212 that were up- and 847 that were down-regulated. (Figure 4B–D). These candidate eccDNAs may play a role in influencing the infection process of BmCPV.

GO analysis was conducted to examine the functions of genes associated with differentially expressed eccDNAs. Genes carried by the up-regulated eccDNAs were involved in biological processes (BP) such as positive regulation of protein secretion, cellular components (CC) such as kinesin complex, and molecular functions (MF) such as microtubule motor activity (Figure 4E–G). Genes carried by down-regulated eccDNAs were involved in BP such as positive regulation of lipid metabolic process, CC such as neuronal cell body, and MF such as phosphatidic acid binding (Figure 4H–J). A total of 72 pathways were found to be up-regulated, including pathways associated with cAMP signaling pathway, PPAR signaling, melanogenesis and others. The 35 down-regulated pathways include those related to pancreatic secretion, morphine addiction, salivary secretion and others (Figure 4J,K). Several eccDNAs contain partial sequences of the SRY-Box transcription factor 6 (SOX6), SoxC transcription factor (sem-2), transcription factors specificity protein 8b (sp8b), and (Foxa2). The findings suggest that these signaling pathways could be involved in the development of the midgut after BmCPV infection.

### 2.5. Association of Differentially Expressed Genes (DEGs) with BmCPV-Infection

**Figure 5:**
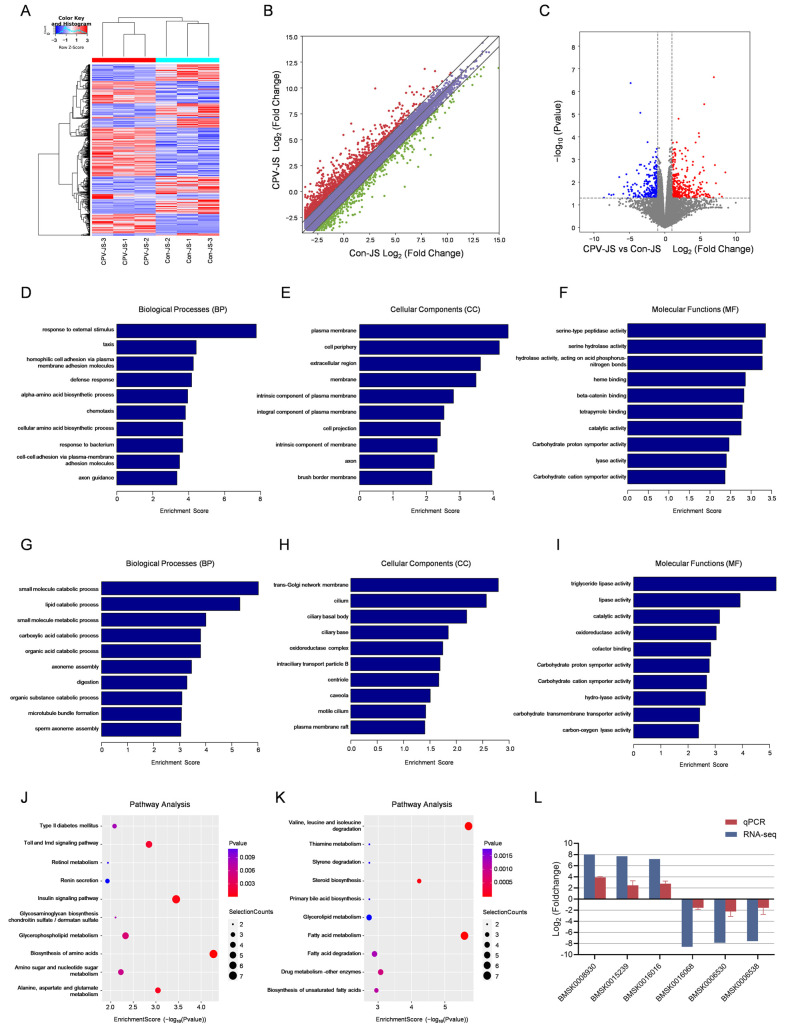
Association of DEGs with BmCPV infection. (**A**) The clustering plot of DEGs. (**B**) The scatter plot of DEGs. (**C**) The volcano plots of DEGs. (**D**–**I**) The biological processes, cellular components, and molecular function related to the up-regulated (**D**–**F**) and down-regulated (**G**–**I**) genes. (**J**,**K**) Analysis of KEGG pathways associated with the up-regulated (**J**) and down-regulated (**K**) genes. (**L**) qPCR validation of RNA-Seq results.

To analyze the gene expression profile in response to BmCPV infection at the whole-genome level, RNA-seq was performed on parallel samples from Circle-seq (Figure 5A–C, Table 2). We identified 562 DEGs, including 316 up- and 246 down-regulated mRNAs in BmCPV-infection samples, compared to BmCPV-uninfected samples. GO terms and KEGG enrichment analyses were performed for these DEGs. Enrichment was observed in GO terms associated with response to external stimulus, plasma membrane, serine-type peptidase activity, small molecule catabolic process, trans-Golgi network membrane, and triglyceride lipase activity (Figure 5D–I). The 17 pathways associated with upregulated genes included biosynthesis of amino acids, insulin signaling pathway, alanine, aspartate and glutamate metabolism, among others (Figure 5J). The 29 pathways associated with downregulated genes included valine and several others (Figure 5K). Overall, following BmCPV infection, significant changes occurred in GO terms and KEGG pathways of DEGs, suggesting that DEGs play a role in the interaction between the virus and the host. To verify the reliability of RNA-seq data, we designed primers for 3 up- and 3 down-regulated DEGs in each treatment based on RNA-seq results. The trends observed in the qPCR results were consistent with those in the RNA-seq data, indicating a high level of credibility for the RNA-seq results (Figure 5L).

### 2.6. Differentially Expressed miRNAs (DEMs) with BmCPV-Infection

**Figure 6:**
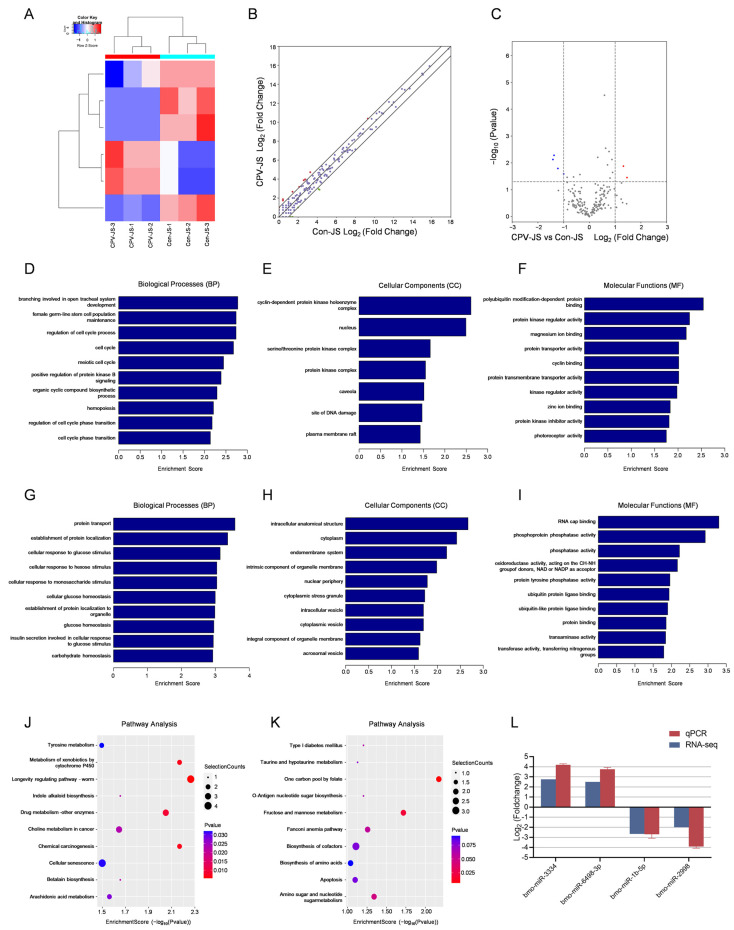
DEMs with BmCPV infection. (**A**) The clustering plot of DEMs. (**B**) The scatter plot of DEMs. (**C**) The volcano plots of DEMs. (**D**–**I**) The biological processes, cellular components, and molecular function related to the up-regulated (**D**–**F**) and down-regulated (**G**–**I**) miRNAs. (**J**,**K**) Analysis of the KEGG pathways associated with the up-regulated (**J**) and down-regulated (**K**) miRNAs. (**L**) qPCR validation of miRNA-Seq results.

EccDNA containing miRNA-1792 notably enhanced the proliferation and migration of HCC (hepatocellular carcinoma) cells in tumors [27]. To verify the correlation between eccDNAs and miRNAs, miRNA-seq was performed on parallel samples from Circle-seq. The sequencing results are outlined in Table 3. A total of 205,942,278 raw reads were collected, with each sample yielding an average of approximately 34 million reads. Each library’s raw reads contained over 90% pure reads, and the average Q30 was 95%. We identified 6 DEMs, comprising 2 that were up-regulated and 4 that were down-regulated (Figure 6A–C). In total, 250 target genes linked to the upregulated miRNAs were discovered. GO analysis (Figure 6D–F) indicated significant enrichment in pathways related to branching involved in open tracheal system development, the cyclin-dependent protein kinase holoenzyme complex, and polyubiquitin modification-dependent protein binding KEGG analysis (Figure 6J) indicated significant enrichment in the longevity regulating pathway-worm, among others. We identified 805 target genes linked to the downregulated miRNAs. GO analysis (Figure 6H–J) revealed significant enrichment in GO terms such as intrinsic components of organelle membranes, oxidoreductase activity and others. KEGG analysis (Figure 6K) indicated significant enrichment in pathways related to one carbon pool by folate and others. To validate the transcriptome results, we utilized stem-loop qPCR to assess whether the expression patterns of these miRNAs matched the results from sequencing. The results revealed that the expression trends of the randomly selected 4 miRNAs matched the sequencing data, confirming the reliability of the small RNA sequencing and bioinformatics analysis techniques (Figure 6L).

### 2.7. Association Analysis of eccDNA-mRNA/miRNA/circRNA

**Figure 7:**
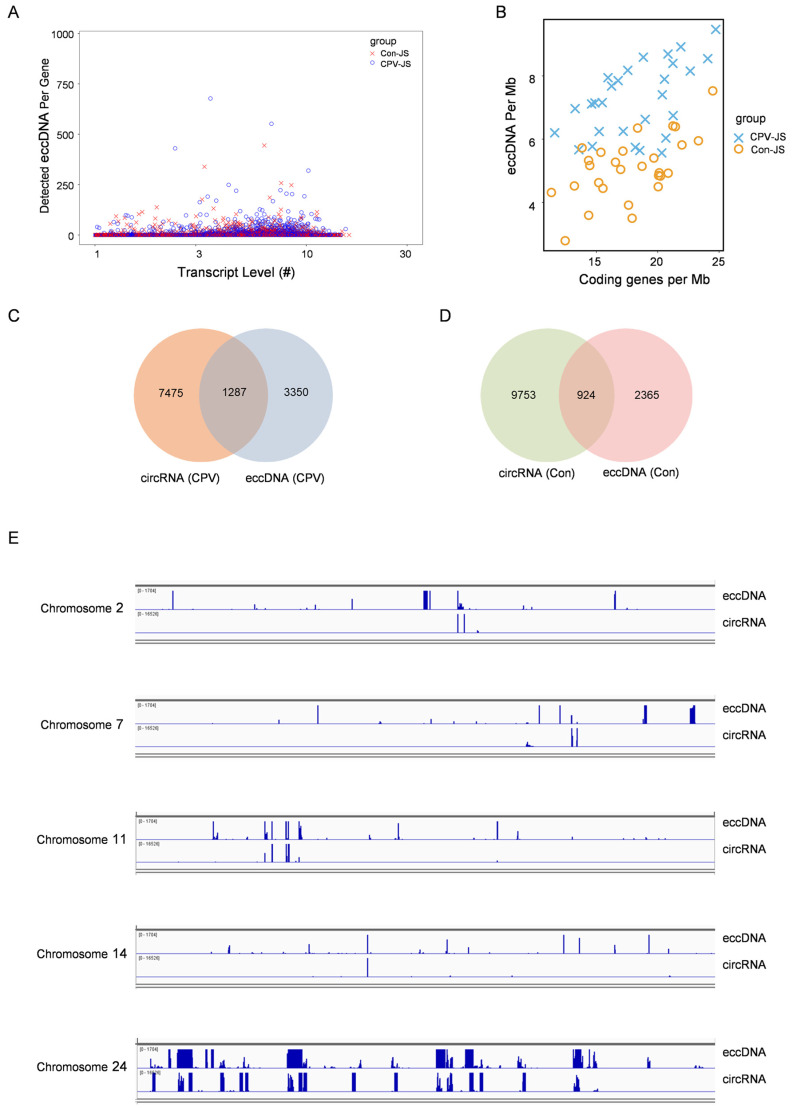
Association analysis of eccDNA-mRNA. (**A**) Association analysis between eccDNA counts in each gene and gene expression profiling. The scatter plot shows the expression levels of individual genes compared to the total expression level of eccDNA linked to each gene. (**B**) eccDNA abundance and gene density distribution. The scatter plot shows the quantity of genes and eccDNAs per MB of length for each chromosome. (**C**,**D**) The sequences representing back-spliced junctions of some circRNAs were shared by junctions of eccDNAs in the BmCPV-infected (**C**) and -uninfected silkworms (**D**). (**E**) Visualization of the genomic locations of eccDNAs and circRNAs loci.

An in-depth analysis was performed to explore the connection between the expression levels of the transcripts from the encoded genes and the frequency of eccDNAs. The results showed that the gene expression level is weakly positively correlated with the eccDNA detected in each gene, but there is no significant difference between the BmCPV infected group and the non-infected group, suggesting that some highly expressed genes are transcribed from relevant sequences of eccDNA, in addition to genomic linear DNA (Figure 7A). A positive correlation existed between the number of eccDNAs detected per megabase pairs (eccDNAs per Mb) and gene density (coding genes per Mb). The ratio of eccDNAs per Mb to coding genes per Mb is higher in the BmCPV-infected group than in the non-infected group (Figure 7B). These findings indicate that the frequency of eccDNA formation is associated with the transcription of coding genes.

As mentioned above, BmCPV infection increased the abundance and quantity of eccDNA in the midgut, with significant changes in the expression levels of 6 miRNAs. However, eccDNA-miRNA association analysis did not identify miRNAs whose expression levels were altered by changes in ecccDNA abundance and quantity.

We previously performed circRNA-seq on the midgut of BmCPV-infected and BmCPV-uninfected silkworms [24]. The combined analysis of the results from eccDNA sequencing and circRNA-seq revealed that 1287 and 924 sequences representing back-spliced junctions of circRNAs were shared by the junctions of eccDNAs in the BmCPV-infected and uninfected silkworms, respectively (Figure 7C,D). Moreover, some eccDNAs loci were found to be shared by circRNAs on Chromosomes 2, 7, 11, 14, and 24 (Figure 7E). These findings further suggest that some eccDNAs may exert its function by being transcribed into circRNAs and changes in the expression levels of some circRNAs may be related to changes in the abundances of eccDNAs after viral infection.

## 3. Discussion

The common occurrence of eccDNAs across various eukaryotic organisms suggests that the ability of genomes to generate and maintain eccDNA is a conserved trait. The data indicate that eccDNAs are present in the midgut of silkworms, further supporting the notion that these molecules are found across different species. The length of eccDNAs identified in the silkworm midgut ranged 72 bp to 17 kb, with most being 100 to 1000 bp. This finding contrasts with results observed in humans and pigeons [5,7]. Through Circle-seq, we identified 35,346 eccDNAs in the silk gland of silkworm [28], while only 5508 eccDNAs were detected in the midgut of silkworm. This difference may be attributed to the developmental characteristics of the silk gland, where cells undergo highly efficient nuclear replication, resulting in a DNA content increase of 2^17^ to 2^19^ times. In contrast, midgut cells do not exhibit such a significant increase in DNA content [29,30]. Most of the eccDNA we detected was in fact microDNA, which is less than 1000 bp long.

The eccDNAs described in this report exhib characteristics (such as length and genomic distribution) that are similar to those of previously characterized eccDNAs [26,31]. Genome-wide eccDNAs in different samples exhibit a higher GC content compared to the average GC content of the genome [31,32,33,34], suggesting that eccDNA formation from the cellular genome is a structured process rather than a random event [31]. However, the GC content of eccDNAs from BmCPV-uninfected silkworms is similar to that of infected silkworms. Cluster analysis of DEGs from eccDNAs, mRNAs, and miRNAs revealed the expression patterns of mRNAs and miRNAs to be more closely aligned with each other across the three replicate samples, while differences in eccDNA expression were more pronounced, this suggests that eccDNA was more heterogeneous in midgut cells. We report the midgut of BmCPV-infected specimens to contain significantly more eccDNAs compared with midguts of uninfected specimens. This phenomenon may be related to induction of apoptosis following BmCPV infection [35]. EccDNA production can be stimulated by apoptosis inducers, which facilitate the fragmentation of apoptotic DNA and its ligation by DNA ligase 3 [36]. The apoptosis induced by BmCPV infection leads to generation of small fragments, which subsequently link to form eccDNA.

The mechanisms underlying the formation of eccDNA have not yet been fully elucidated, and various potential mechanisms for the generation of eccDNA have been proposed [15,31]. Most eccDNAs originate from repetitive sequences on chromosomes, homologous recombination or MMEJ between short repeat sequences may lead to its production [37,38,39,40,41,42]. Nevertheless, a substantial fraction of eccDNA lacks any repeat sequences, making it unable to recombine with nearby sequences [39]. The level of eccDNA may depend on excision following double-strand breaks and the repair through MMEJ [43]. It is unknown if the process of DNA replication contributes to the generation of eccDNA. Levels of eccDNA are increased when ongoing replication is blocked by replication inhibitors [40], but eccDNA can also be generated without DNA replication [44]. Additionally, it has been found that apoptosis also promotes the formation of eccDNA [36]. Consistent with previous studies [26,45], we observed a similar short direct repeat surrounding the eccDNA molecules. Regions where eccDNAs are formed are not randomly distributed within chromosomes. EccDNAs are derived from thousands of unique sites across the genome and are particularly enriched in regions known as hotspots [5]. Our findings contradict this model, as the flanking sequences exhibit a high AT content. Our results are, however, consistent with observations made in silkworm silk glands [28]. This indicates that eccDNA formation is not dependent on a single mechanism, but the formation pattern in the silkworm may be consistent.

EccDNA’s function is significantly related to the genes it carries. GO and KEGG analysis reveals most eccDNAs carrying genes involved in microtubule motor activity, phosphatidic acid binding, cAMP signaling pathway, and pancreatic secretion signaling pathways, which may play a regulatory role in BmCPV infection. Research indicates that eccDNAs carrying Alu-like sequences are likely to be transcribed [46,47]. Yerlici et al. demonstrated that an eccDNA from *Oxytricha* can be transcribed to produce specific lncRNA [6]. This indicates that eccDNAs may function through transcription to generate RNAs. Through the integration analysis of eccDNA and mRNA, the transcriptional activity of coding genes have been demonstrated to influence the frequency of eccDNA formation. Additionally, we found 5 eccDNAs containing partial sequences of SOX6, two eccDNAs containing partial sequences of the transcription factor sem-2, and one eccDNA containing partial sequences of sp8b and Foxa2. As members of a superfamily of transcription factors, SOX proteins display characteristics associated with both classical transcription factors and the structural components of chromatin. SOX6 inhibits proliferation of cervical cancer cells by inducing cellular senescence [48,49]. The regulation of twist-related protein (hlh-8) expression by SEM-2 seems to be direct; hlh-8 encodes a basic helix-loop-helix twist transcription factor that plays a crucial role in the proper patterning of the M lineage [50]. The knockdown of Foxa2 results in the upregulation of genes involved in the phosphatidylinositol 3-kinase (PI3K)/Akt pathway and activates downstream phosphorylation of Akt [51]. The expression of Foxa2 and nuclear receptor subfamily 4 group A member 2 (Nurr1) mediated by adeno-associated virus can alleviate cognitive deficits in mice with Alzheimer’s disease [52]. Following BmCPV infection, the expression levels of these transcription factor-carrying eccDNAs were up-regulated, suggesting that they may function through transcription.

The small size of eccDNAs (<1000 bp) limits their ability to carry complete protein-coding genes, however, their length is sufficient to accommodate complete miRNA genes. Paulsen et al. investigated whether eccDNAs without a typical promoter could be transcribed in mammals and whether the resulting transcription products were functional in cells. The results indicate that some eccDNAs can be transcribed to produce RNAs with regulatory functions, including miRNAs and novel siRNAs. These findings offer a novel mechanism for comprehending the genomic plasticity and instability that result in alterations in gene expression [18]. In this study, after BmCPV infection, eccDNA up-regulation increased significantly, however, we did not identify miRNAs whose expression levels were altered by changes in ecccDNA abundance and quantity. In our previous study, 9753 and 7475 circRNAs were identified in the midgut of healthy silkworm and BmCPV-infected silkworm, of which 294 circRNAs were up-regulated and 106 circRNAs were down-regulated following infection, and 737 circRNAs were expressed specifically in the BmCPV-infected silkworm [24]. In ths study, it was found that the sequences representing back-spliced junctions of some circrNAs were shared by junction sequences of some eccDNAs and some eccDNAs loci were shared by circRNAs, suggesting that some eccDNAs may be transcribed to form circRNAs and changes in the expression levels of some circRNAs may be related to changes in the abundance of eccDNA after viral infection.

In conclusion, this study indicates the presence of a large amount of eccDNA in the midgut of silkworms, with most eccDNA ranging in size from 100 to 1000 bp, and some eccDNA containing sequences of transcription factors. After infection with BmCPV, the abundance of eccDNA increases. Some eccDNAs may regulate viral replication by transcribing into mRNA, miRNA, or circRNA. These findings deepen our understanding of the interaction mechanism between BmCPV and silkworms from a new perspective. It should be pointed out that the results obtained in this study mainly rely on bioinformatics analysis of high-throughput sequencing data. In the future, further experiments are needed to verify the association between eccDNA-mRNA/miRNA/circRNA and its function in BmCPV infection.

## 4. Materials and Methods

### 4.1. Sample Preparation

The silkworm (Jingsong strain) used in this study were provided by Jiangsu Province Silkworm Germplasm Resources Protection Bank (Suzhou, China). BmCPV stock solution was prepared following our previous study [53]. The silkworms on the 1st day of 3rd instar were fed with the mulberry leaves coated with the BmCPV polyhedra suspension (1 × 10^8^/mL, 1 mL) for 8 h, after which they were fed fresh mulberry leaves. The midguts were collected at 3rd day 5th instar and named BmCPV-infected. The silkworms on the 1st day of 3rd instar were fed with the normal mulberry leaves coated with water as control. After 8 h feeding, they were given fresh mulberry leaves. The midguts were collected at the 3rd day of 5th instar and named BmCPV-uninfected. For each sample, three replicates were established.

### 4.2. Circle-Seq

Circle-seq was conducted by CloudSeq Biotech Inc. (Shanghai, China) following the methods described in our previously report [28]. The data processing and analysis obtained from circle-seq were also conducted according to our previous research. All sequencing data have been uploaded to the NCBI database (SRR31210857–62).

### 4.3. Validation of eccDNAs

Outward-directed PCR primers were employed to validate the junction site of the detected eccDNAs (Table 4). The PCR reaction was conducted according to the manufacturer’s instructions (Takara, Japan). The PCR products were analyzed using 1% agarose gel electrophoresis. Subsequently, the recovered products were cloned into the pMD-19T vector (TaKaRa, Dalian, China) for Sanger sequencing.

### 4.4. Motifs of eccDNA Breaking Points

For each eccDNA locus, the nucleotide sequence of the 10 bp flanking the 3′ and 5′ breaking points were analyzed. Using computer simulation, we computed the expected frequency of each motif, and new positions in the genome for each eccDNA length were randomly assigned using WebLogo (http://weblogo.berkeley.edu/logo.cgi) (accessed on 1 December 2023).

### 4.5. GO and KEGG Analyses

GO (http://www.geneontology.org) (accessed on 1 December 2023) and KEGG (http://www.genome.jp/kegg/) (accessed on 1 December 2023) were used to annotate the gene functions [24]. we extracted eccDNAs containing full or partial genes for GO and KEGG enrichments (*p* ≤ 0.05). The Benjamini-Hochberg (BH) approach was employed to determine correct *p*-values [54].

### 4.6. RNA-Seq Analysis

Total RNA was extracted from both BmCPV-infected and uninfected silkworm midguts, utilizing the same samples as in the Circle-Seq analysis. Library preparation and RNA sequencing were conducted on an Illumina HiSeq 4000 sequencer. Paired-end reads were obtained from the sequencer and underwent quality control with Q30. Following 3′ adaptor trimming and the removal of low-quality reads using Cutadapt (v1.9.3) [55], high-quality clean reads were aligned to the reference *B. mori* genome using HISAT2 (v2.0.4) (http://ccb.jhu.edu/software/hisat2/index.shtml) (accessed on 19 November 2023). Raw counts were generated using HTSeq (v0.9.1) [56], and normalization was performed with edgeR [57]. DEGs were identified based on *p*-value < 0.05 and log_2_|fold change| > 1. Annotation of DEGs was conducted using GO and KEGG pathway. All sequences have been uploaded to the NCBI database (SRR31214593–98).

### 4.7. miRNA-Seq Analysis

Total RNA was extracted from both BmCPV-infected and uninfected silkworm midguts, utilizing the same samples as in the Circle-Seq analysis. The small RNA library was prepared using the GenSeq Small RNA Library Prep Kit (GenSeq, Inc.) (Nanjing, China) following manufacturer instructions, and then sequenced on an Illumina HiSeq sequencer. Subsequently, the raw reads were collected following quality control with Q30. Adapter sequences were removed from the original reads using Cutadapt [55], and low-quality reads were filtered out, retaining only those ≥ 15 nt in length to obtain trimmed reads. These trimmed reads from all samples were merged, and new miRNAs were predicted using miRDeep2 (v2.0.0.5) [58]. Each sample’s trimmed reads were aligned to the merged pre-miRNAs database (miRBase [http://www.mirbase.org] (accessed on 15 November 2023), v22 pre-miRNAs + newly predicted pre-miRNAs) using Novoalign (v3.02.12) (https://www.novocraft.com/products/novoalign/) (accessed on 15 November 2023); 1 mismatch was allowed. The raw expression of each mature miRNA was determined by counting the number of tags aligned to it, which were then normalized using the TPM method [59]. *p*-value < 0.05 and log_2_|fold change| > 1 were used to screen DEMs. Target genes of known DEMs were predicted using miRNA target gene prediction softwares Miranda (https://www.miranda.software/, accessed on 9 January 2024) [60] and TargetScan ( https://www.targetscan.org/vert_80/, accessed on 9 January 2024) [61]. The resulting miRNA-target gene network was created with Cytoscape (v2.8.0) [62]. The target genes were subjected to GO and KEGG enrichment analysis. All sequences have been uploaded to the NCBI database (SRR31215093–98).

### 4.8. Association Analysis of eccDNA-miRNA/circRNA

The association analysis of eccDNA-miRNA/circRNA was conducted by CloudSeq Biotech Inc. (Shanghai, China). Briefly, the location of differentially expressed miRNAs precursors in the genome (https://silkdb.bioinfotoolkits.net/main/species-info/-1) (accessed on 9 January 2024) of silkworm was obtained by searching the microRNA database (https://www.mirbase.org/browse/results/?organism=bmo) (accessed on 9 January 2024). If the precursor sequence of a differentially expressed miRNA is completely covered by the genomic region where a differentially expressed eccDNA is located, it is considered that the eccDNA has the potential to encode miRNA, and thus, an association between eccDNA-miRNA is established.

The back-spliced junction site of circRNA and the junction site of eccDNA were analyzed using CIRCexplorer2 [63]. The location of circRNA and eccDNA were visualized using IGV (v2.4.10) software [64]. If the sequence of a differentially expressed circRNA is completely covered by the genomic region where a differentially expressed eccDNA is located, it is considered that the eccDNA has the potential to encode circRNA. Calculate the expression level (expressed as reads) of circRNA on the eccDNA locus using the multibigwigSummary command in DeepTools software (https://deeptools.readthedocs.io/en/develop/, accessed on 9 January 2024). When the reading value of the effective reading contained in the bigwig file of the circRNA on a specific eccDNA locus is greater than 0, it is considered that the circRNA located on the corresponding eccDNA is expressed.

### 4.9. RNA Extraction and qPCR Analysis

To determine the expression levels of DEGs and DEMs, total RNA was extracted from the midguts of both BmCPV-infected and uninfected silkworms and reverse transcribed into cDNA (TransGen Biotech, Beijing, China). The relative transcriptional levels were determined by real-time PCR using the primers listed in Table 4. The housekeeping gene *TIF-4A* was used as normalization.

### 4.10. Western Blot

Total protein (40 µg/lane) of midguts was separated by SDS-PAGE and subsequently transferred to the PVDF membrane (Millipore, Burlington, MA, USA). After blocking the membranes with 3% BSA in PBST containing 0.05% Tween-20, Western blot was performed by using mouse anti-VP1 as the primary antibodies. The secondary antibodies used were HRP-labeled goat anti-mouse IgG (Proteintech, Chicago, IL, USA).

### 4.11. Statistics

Data are presented as means ± standard deviation (SD). Statistical analyses were conducted using one-way analysis of variance (ANOVA) and *t*-test to assess significance between groups, employing GraphPad Prism 8 software. *p*-value significance was set at *p* ≤ 0.05.

## Figures and Tables

**Table 1:** The summary of reads and in each sample.

Sample	Raw Reads	Clean Reads	Mapped Reads	Mapped Rate
CPV-JS-1	221,627,248	221,622,188	168,621,706	76.09%
CPV-JS-2	196,407,022	196,398,878	152,921,996	77.86%
CPV-JS-3	153,286,150	153,274,728	116,076,328	75.73%
Con-JS-1	148,156,952	148,146,262	120,498,260	81.34%
Con-JS-2	146,219,962	146,211,074	111,350,696	76.16%
Con-JS-3	137,831,542	137,778,670	75,362,596	54.70%

**Table 2:** The summary of reads in each sample.

Sample	Raw Reads	Clean Reads	Mapped Reads	Mapped Rate
CPV-JS-1	47,659,218	47,513,488	38,211,453	80.42%
CPV-JS-2	46,644,662	46,533,786	37,816,326	81.27%
CPV-JS-3	40,258,640	40,161,644	30,718,884	76.49%
Con-JS-1	50,932,130	50,791,626	40,712,037	80.16%
Con-JS-2	50,553,920	50,450,864	39,448,288	78.19%
Con-JS-3	56,244,622	56,113,548	44,780,534	79.80%

**Table 3:** The summary of reads in each sample.

Sample	Raw Reads	Mapped Reads	Mapped Rate	Q30
CPV-JS-1	38,306,407	35,285,596	92.11%	96.03%
CPV-JS-2	36,566,873	33,470,524	91.53%	96.21%
CPV-JS-3	31,781,525	28,761,924	90.50%	95.38%
Con-JS-1	37,520,823	34,732,059	92.57%	95.94%
Con-JS-2	33,285,246	30,442,799	91.46%	96.12%
Con-JS-3	28,481,404	26,107,336	91.66%	95.94%

**Table 4:** Primer sequence.

Name	Forward Primer	Reverse Primer
eccDNA-1	AGTACGTCACCTACCTTCGCA	CACAGGTACTGCACAAAGGAA
eccDNA-2	GCTACCAGCGTATAGGTAGGC	CCTACCCATAATACCGCGCC
eccDNA-3	AATGGCTTCACTTGTAGCACG	GGGCGGGTTCGGATAATCA
eccDNA-4	AAGAGCCTCACAGAGTGCTT	CGCGAGTAGCGTTCAATGAT
eccDNA-5	GCTCGTCGGTCAAATAATGCT	GATTCGGCCACAACCGAAAT
BMSK0008930	ACGAAGGATCCCTTTCCGTT	AGGCCTCTACGTCCATCAAG
BMSK0015239	CCTGTTCAGCAGAAGGAGGA	TGGAAGCCAATGAAGGCAAC
BMSK0016016	GTCTTCGGGACTTTGGGAGA	TAGACGACCACCGTAACTGG
BMSK0016068	CAGGAACAGGCTCTTCTGGA	GACCAAGCGTATTCGTAGCC
BMSK0006530	AGCGCGTTCTATTCATGCAG	TTCCTGCAGGTAAGGTTGGT
BMSK0006538	CCCTTGGGCAACTACAGAGG	CGAGAGCTTTGCTGGTGTAG
RT-miR-3334	CTCAACTGGTGTCGTGGAGTCGGCAATTCAGTTGAGCTGTCCTT	
RT-miR-6498-3p	CTCAACTGGTGTCGTGGAGTCGGCAATTCAGTTGAGAACTGTAT	
RT-miR-1b-5p	CTCAACTGGTGTCGTGGAGTCGGCAATTCAGTTGAGTATGGAAT	
RT-miR-2998	CTCAACTGGTGTCGTGGAGTCGGCAATTCAGTTGAGTTTATCTA	
bmo -miR-3334	ACACTCCAGCTGGGTGAACCAGAATGATGGAA	CTCAACTGGTGTCGTGGAGTCG
bmo -miR-6498-3p	ACACTCCAGCTGGGAACGTCTGCGATGAT	CTCAACTGGTGTCGTGGAGTCG
bmo -miR-1b-5p	ACACTCCAGCTGGGCCATACTTCTTTACAT	CTCAACTGGTGTCGTGGAGTCG
bmo-miR-2998	ACACTCCAGCTGGGAAGAACAGGATGAGGTA	CTCAACTGGTGTCGTGGAGTCG

## Data Availability

Data are contained within the article.
